# Population genetic analysis of autophagy and phagocytosis genes in *Drosophila melanogaster* and *D*. *simulans*

**DOI:** 10.1371/journal.pone.0205024

**Published:** 2018-10-03

**Authors:** Joo Hyun Im, Brian P. Lazzaro

**Affiliations:** 1 Cornell Institute of Host-Microbe Interactions and Disease, Cornell University, Ithaca, NY, United States of America; 2 Graduate Field of Genetics, Genomics, and Development, Cornell University, Ithaca, NY, United States of America; 3 Department of Entomology, Cornell University, Ithaca, NY, United States of America; University of Iceland, ICELAND

## Abstract

Autophagy and phagocytosis are cellular immune mechanisms for internalization and elimination of intracellular and extracellular pathogens. Some pathogens have evolved the ability to inhibit or manipulate these processes, raising the prospect of adaptive reciprocal co-evolution by the host. We performed population genetic analyses on phagocytosis and autophagy genes in *Drosophila melanogaster* and *D*. *simulans* to test for molecular evolutionary signatures of immune adaptation. We found that phagocytosis and autophagy genes as a whole exhibited an elevated level of haplotype homozygosity in both species. In addition, we detected signatures of recent selection, notably in the *Atg14* and *Ykt6* genes in *D*. *melanogaster* and a pattern of elevated sequence divergence in the *genderblind* (*gb*) gene on the *D*. *simulans* lineage. These results suggest that the evolution of the host cellular immune system as a whole may be shaped by a dynamic conflict between *Drosophila* and its pathogens even without pervasive evidence of strong adaptive evolution at the individual gene level.

## Introduction

**Fig 1:**
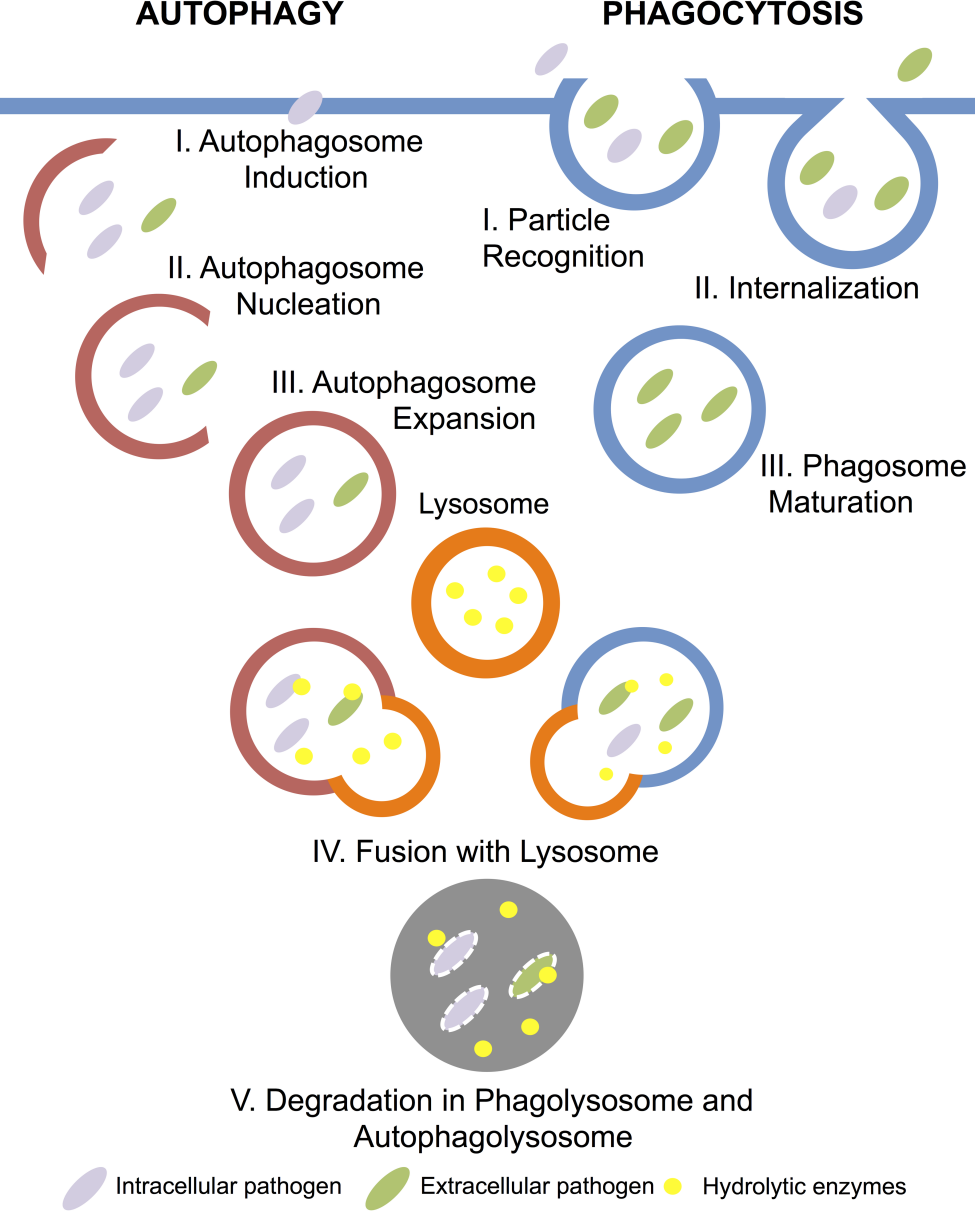
Stages of autophagy and phagocytosis pathways. Genes in autophagy (red) and phagocytosis (blue) pathway function to recognize, internalize, and degrade cell debris and intracellular (purple) and extracellular pathogens (green). Organelles, such as phagosomes and autophagosomes, are form in the course of the process and are eventually fused with a lysosome full of hydrolytic enzymes (yellow) to degrade internalized pathogens.

Phagocytosis is a primary cellular immune process in *Drosophila* [1]. During phagocytosis, extracellular pathogens are recognized by opsonins and phagocytic receptors, engulfed at the host membrane, and then internalized and degraded in compartments called phagosomes [2] (Fig 1). Autophagy is an alternative cellular mechanism to remove intracellular pathogens [3]. During autophagy, intracellular bacteria and viruses are encapsulated by isolation membranes called phagosphores, which then are nucleated and expanded to form autophagosomes to destroy the pathogen [4, 5] (Fig 1). Both phagosomes and autophagosomes are eventually fused with a lysosome to degrade internalized pathogens [6]. While autophagy and phagocytosis were previously thought to be distinct pathways, many autophagy proteins participate in the later stages of phagocytosis [6–8]. When phagocytosis fails to eliminate pathogens due to modification or damage to the phagosome by bacteria, autophagy works as a back-up process to overcome infection [9, 10].

Pathogens evolve to escape, resist, or compromise the host immunity [11]. Bacteria are known to inhibit or evade phagocytosis by preventing host opsonins and phagocytic receptors from binding to bacterial molecules [12, 13] and blocking host signaling pathways via effector proteins and toxins [14–16]. Similarly, bacteria and viruses also interfere with host autophagy by disrupting the signaling [17, 18] and blocking the production of reactive oxygen species (ROS) that sustains autophagy [19]. When a pathogen factor interferes with a critical host protein, the host protein can counter-evolve the bacterial hindrance via novel mutations. The change in the host may place renewed evolutionary pressure on the bacteria, and the process can repeat *ad infinitum* leading to a dynamic co-evolutionary conflict, or arms race [20, 21]. Host alleles that resist or overcome pathogen interference mechanisms may be adaptive, potentially resulting in signatures of positive selection as beneficial alleles favored by natural selection rise to high frequency within a population. These alleles would exhibit a reduced level of polymorphism around the selected sites, and would accumulate a proportional excess of rare variants as the population recovers from a selective sweep [22]. Recurrent adaptive fixations over long evolutionary timescales could result in an elevated rate of amino acid divergence between species [23].

In this study, we used molecular population genetic analyses to test for adaptation in autophagy and phagocytosis genes. We analyzed published sequence data from *D*. *melanogaster* and *D*. *simulans* collected in Eastern Africa [24, 25]. We found that genes in autophagy and phagocytosis pathways as a whole showed an elevated level of homozygosity in both species. The individual genes *Atg14* and *Ykt6* showed evidence of non-neutral evolution in *D*. *melanogaster* while *gb* showed signatures of non-neutral evolution in *D*. *simulans*.

## Materials and methods

### Samples used in the population genetic analysis

For *D*. *melanogaster*, the sequenced genomes of 197 haploid embryo Siavonga lines from the *Drosophila* Genome Nexus Project 3 were used [25]. These lines represent a single ancestral population of *D*. *melanogaster* from Zambia. For *D*. *simulans*, genome sequences from 20 isofemale lines, 10 collected in Madagascar and 10 collected in Kenya, were used [24]. In addition, a reference sequence of *D*. *yakuba* was used as an outgroup [26].

### Curating genes of interest and control genes from the literature

A list of genes known to be involved in phagocytosis and autophagy was established by reviewing the primary literature (S1 and S2 Tables). In addition to the known phagocytosis and autophagy genes in *Drosophila*, *Drosophila* homologs of phagocytosis or autophagy genes characterized in other organisms were included. Each gene was assigned to a functional category (e.g. Autophagy induction) (S1 and S2 Tables; Fig 1). To test whether phagocytosis and autophagy pathways show different evolutionary patterns than canonical humoral pathways, known humoral immune signaling and recognition genes were used as a comparison. To control for effects of gene structure and chromosomal position, control genes were chosen to be similar to the focal immune genes in gene length (0.5-2x length of the coding region of the focal gene) and gene location (within 60kb ± of the start site of the focal gene). Three or four control genes that matched the criteria and had no annotated immune function were chosen for each focal gene. If a focal gene had fewer than three control genes that matched the criteria, it was removed from downstream analysis. Pairing focal and control genes controlled for potential effects of gene length and recombination rate. To ensure that focal and control genes indeed are matched for local recombination rate, the predicted recombination rates for all genes were examined using the *Drosophila melanogaster* Recombination Rate Calculator (RRC, version 2.3, [27]). This tool provides estimates of recombination rates based on the Marey map approach [27] and direct measurement [28]. Both RRC and Comeron estimates showed that the correlation between recombination rates of focal and control genes in *D*. *melanogaster* is strong, confirming that focal and control genes truly share a similar recombination environment (R^2^ = 0.994 based on RRC midpoint and R^2^ = 0.890 based on Comeron midpoint, S1 Fig). The presence of inversions can create strong haplotype structure and influence patterns of polymorphisms [29]. To minimize the effect of inversions, no focal gene or control gene used in the study was near the boundaries of known inversions. Populations collected in Africa sometimes contain genomic segments inferred to have recent cosmopolitan (non-African) ancestry [30]. To specifically analyze African genetic variation and eliminate the effect of relatedness among individual lines, genomic regions that were thought to have come from cosmopolitan (non-African) ancestry and that showed evidence of identity-by-descent in *D*. *melanogaster* were masked using Perl scripts obtained from http://www.johnpool.net/genomes.html.

### Processing sequence data prior to population genetics analyses

Using custom scripts and bedtools [31], the coding sequence of each gene was extracted based on coordinates from the General Feature Format (GFF) file of the reference sequence for *D*. *melanogaster* (FlyBase release 5.25) and from the GFF file provided by the Thornton Lab GitHub (https://github.com/ThorntonLab) for *D*. *simulans*. When multiple isoforms were available for a gene, the longest sequence was chosen for downstream analysis. Custom filters were applied to exclude sequences with sites containing more than 10% missing data (noted as Ns) and then alignment was performed using PRANK [32]. Genes with poor sequencing or alignment quality, with large regions of gap, or with no annotation in any of the species were excluded from downstream analysis. To standardize the number of lines surveyed for each gene, 149 *D*. *melanogaster* lines and 14 *D*. *simulans* lines were randomly subsampled.

### Surveying the level of polymorphisms and divergence

To evaluate the patterns of polymorphisms in each species, the following population genetic statistics were calculated: Watterson’s θ (θ_*w*_), Tajima’s *D* (TajD), normalized Fay and Wu’s *H* (nFWH), Ewens-Watterson test statistic (*EW*), and the compound statistics that combined Tajima’s *D*, normalized Fay and Wu’s *H* with Ewens-Watterson test statistic (*DHEW*). θ_*w*_ indicates the level of DNA sequence variation, and a reduction in sequence diversity can be due to a recent selective sweep [33]. Tajima’s *D* test statistic compares the number of pairwise differences between individuals to the total number of segregating sites and detects the level of mutations of intermediate frequency relative to mutations that segregate at low frequencies [34]. The mutations that initially arise after variation is purged in a selective sweep will necessarily be at low frequency, skewing the site frequency spectrum and causing Tajima’s *D* to become negative [23]. Negative Tajima’s *D* values can also be caused by population expansion. Fay and Wu’s *H* test statistic detects excess of high frequency alleles in the derived state, which is a signature of selective sweeps that is less likely under population expansion [35]. The normalized version of the *H* test was used to increase statistical power. The *EW* test statistic measures haplotype homozygosity by comparing the observed homozygosity to the expected homozygosity [36, 37]. A P-value greater than 0.95 suggests that allele frequencies are more unevenly distributed than the neutral expectation, which suggests directional selection. The *DHEW* statistic is a composite statistic that provides more power to detect cases of positive selection [38, 39]. By combining Tajima’s *D* and normalized Fay and Wu’s *H* with Ewens-Wattersons expected homozygosity (*EW*), which is generally not sensitive to recombination, the *DHEW* overcomes the effect of recombination rates on the site frequency spectrum and makes the inference more conservative. The *DHEW* test first calculates required statistics separately and then combines the respective P-values into a vector to determine whether this vector deviates from the expectation under selective neutrality [38, 39]. A significant P-value points to positive selection. To calculate aforementioned statistics, the program DH was used [38–40]. Each test statistic was compared to 1) null distributions created using coalescent simulations with no recombination to obtain a P-value [39], and 2) the test statistics obtained from genomic control genes.

To evaluate the patterns of amino acid divergence, the ratio of non-synonymous to synonymous polymorphic sites in each species (Pn/Ps) and the ratio of non-synonymous to synonymous differences between the species (Dn/Ds) were calculated and the McDonald-Kreitman test (MK test) was performed [41] using a custom script. If the observed value of Pn/Ps is much different from Dn/Ds at a locus as determined by Fishers exact test, that locus is rapidly diverging between the two species at the level of amino acid sequence, which is consistent with adaptive evolution. In addition, the Direction of Selection (DoS) coefficient was calculated [42]. DoS >0 indicates the action of positive selection and DoS <0 indicates the action of purifying selection. To correct for multiple testing, the p.adjust function in R based on the Benjamini and Hochberg method was implemented [43].

### Processing statistics and testing for significance

To assess whether phagocytosis and autophagy pathways as a whole show a departure from their control genes for various test statistics, the difference between the value of a given statistic in the focal gene and the median value of the statistic for the matched control genes, hereafter called a ‘comparison score’, was calculated for each focal-control pairing. We then evaluated whether the mean of all comparison scores was significantly different from 0 using both a t-test (parametric) and the Wilcox rank sum test (non-parametric) (Table 1). If the 95% confidence interval contains 0, the test provides no evidence for any statistically significant difference between the two groups as a whole. To identify individual genes that bear patterns of non-neutral sequence evolution in each species, the P-values of, *D*, *H*, *EW*, and the compound test statistic *DHEW* were used. To ensure that the pattern seen on focal genes is indeed due to a local selection, the comparison score was calculated for Tajima’s *D*, Fay and Wu’s *H*, and DoS for each focal-control pair and the scores were ranked from largest to smallest (listed as ‘rank’ column in Tables 2 and 3). A total of 68 and 65 comparison values make up the distributions for *D*. *melanogaster* and *D*. *simulans*, respectively. Randomly assigning a control gene to be "focal" and re-calculating the ranks of comparison scores for these statistics created a distribution of ranks. Then comparison scores from true coupling of focal and control genes were compared to this permutated distribution (listed as ‘rank against null’ column in Tables 2 and 3). A total of 245 and 232 values were used to build null distributions for *D*. *melanogaster* and *D*. *simulans*, respectively. The more extreme this rank is, the stronger the confidence is that the focal gene differs from the control genes for a given statistic. We set a threshold for concluding evidence that a gene had experienced a selective sweep as requiring 1) a significant P-value for DHEW, 2) significant corrected P-values for two of any of the test statistics, and 3) comparison scores for *D*, *H*, or DoS ranking in the top 10% for the ‘rank’ test and the top 5% for the ‘rank against null’ test. Individual genes that have potentially undergone adaptive divergence on each lineage were identified as having a statistically significant (P <0.05) MK test result and a Direction of Selection (DoS) coefficient >0.

**Table 1:** Evaluation of polymorphism and divergence at the pathway level.

**I) t-test**					
	***D*. *melanogaster***				
			**Test statistic**		
**Class**	θ_w_	**TajD**	**nFWH**	**EW**	**DoS**
**Internalization**	0.6341	0.0583	-0.0674	**0.0521**	-0.0168
** **	(-2.6688, 3.9369)	(-0.0586, 0.1752)	(-0.2697, 0.1349)	**(0.0079, 0.0962)**	(-0.0589, 0.0252)
	0.7028	0.3233	0.5085	**0.0215**	0.4272
**Signaling**	-1.527795	0.02134113	-0.1812032	0.02269972	0.03661695
	(-4.7315, 1.6759)	(-0.2696, 0.3123)	(-0.5179, 0.1555)	(-0.0496, 0.0950)	(-0.0515, 0.1247)
	0.3334	0.8805	0.2764	0.5217	0.3951
**Recognition**	2.484337	-0.04183397	-0.3390658	-0.009098689	0.006212503
	(-2.2566, 7.2253)	(-0.39654, 0.3127)	(-1.2406, 0.5625)	(-0.0857, 0.0675)	(-0.2837, 0.2961)
	0.2471	0.7825	0.3929	0.781	0.9554
	***D*. *simulans***				
			**Test statistic**		
**Class**	**θ**_**w**_	**TajD**	**nFWH**	**EW**	**DoS**
**Internalization**	-0.5781	0.0174	-0.0506	**0.0587**	-0.0203
** **	(-4.8929, 3.7367)	(-0.1116, 0.1463)	(-0.1939, 0.0927)	**(0.0213, 0.0961)**	(-0.0796, 0.0389)
	0.7898	0.7889	0.4828	**0.0026**	0.4939
**Signaling**	-1.909174	0.004127492	-0.04127675	-0.008017493	0.06485741
	(-7.1512, 3.3329)	(-0.2052, 0.2135)	(-0.3457, 0.2632)	(-0.0312, 0.0152)	(-0.0833, 0.2131)
	0.4563	0.9676	0.7802	0.479	0.3687
**Recognition**	0.9826631	0.03899865	-0.2613483	0.008290816	**-0.1259514**
	(-6.6833, 8.6486)	(-0.3190, 0.3969)	(-0.7113, 0.1886)	(-0.0260, 0.0426)	**(-0.1647, -0.0872)**
	0.7706	0.8041	0.2119	0.5852	**0.0051**
**II) Wilcox rank sum test**					
	***D*. *melanogaster***				
			**Test statistics**		
**Class**	**θ**_**w**_	**TajD**	**nFWH**	**EW**	**DoS**
**Internalization**	-1.0757	0.0451	-0.0031	0.0146	-0.0208
	(-2.8685, 7.8884)	(-0.0634, 0.1600)	(-0.1889, 0.1821)	(-0.0003, 0.0422)	(-0.0640, 0.0264)
** **	0.3552	0.3773	0.9927	0.0554	0.351
**Signaling**	-0.8068	0.0061	-0.1739	-0.0014	0.0484
** **	(-3.8097, 1.7032)	(-0.2858, 0.2906)	(-0.5078, 0.1103)	(-0.0170, 0.0128)	(-0.0604, 0.1234)
	0.4452	0.9881	0.2345	0.7998	0.33
**Recognition**	2.4427	-0.0111	-0.2327	-0.0114	0.0183
	(-2.8685, 7.8884)	(-0.4286, 0.2887)	(-1.3622, 0.5960)	(-0.0851, 0.0586)	(-0.3471, 0.2575)
	0.2969	1	0.375	0.6875	1
	***D*. *simulans***				
			**Test statistics**		
**Class**	**θ**_**w**_	**TajD**	**nFWH**	**EW**	**DoS**
**Internalization**	-2.2012	-0.0254	-0.00345	**0.0153**	-0.0258
	(-5.5030, 1.5722)	(-0.1224, 0.0989)	(-0.1687, 0.1375)	**(0.0026, 0.0944)**	(-0.0840, 0.0396)
** **	0.25	0.6902	0.9375	**0.0053**	0.4788
**Signaling**	-2.437	-0.0239	-0.0657	-0.0052	0.0076
** **	(-7.1538, 2.2012)	(-0.2393, 0.2191)	(-0.3032, 0.2355)	(-0.0230, 0.0204)	(-0.0866, 0.1765)
	0.2157	0.9187	0.5392	0.4542	0.9323
**Recognition**	1.0613	0.0682	-0.1791	0.0127	-0.1245
	(-7.7041, 10.2197)	(-0.3824, 0.3882)	(-0.7856, 0.1794)	(-0.0255, 0.0535)	(-0.1436, -0.1142)
	0.8438	0.7422	0.3125	0.4606	0.25

θ_w_, Watterson’s *θ*; TajD, Tajima’s *D*; nFWH, normalized Fay and Wu’s *H*; EW, Ewens-Watterson statistic; DoS, Direction of Selection; For a given statistic, each value represents the mean (t-test) or pseudomedian (Wilcox) of comparison scores for each focal-control gene pairing and the values in parenthesis are the 95% confidence intervals with an associated P-value below. Significant deviation is bolded.

**Table 2:** Genes that show significant and nominal evidence of recent selection in *D*. *melanogaster* and in *D*. *simulans*.

***D*. *melanogaster***																		
**Gene ID**	**Gene name**	**Function**	**θ**_**w**_	**TajD**	**TajD pval**	**TajD pval corrected**	**nFWH**	**nFWH pval**	**nFWH pval corrected**	**EW**	**EW pval**	**EW pval corrected**	**DHEW pval**	**DHEW pval corrected**	**TajD rank**	**TajD rank against null**	**nFWH rank**	**nFWH rank against null**
**FBgn0039636**	***Atg14***	**Autophagy: Nucleation**	**9.1430**	**-1.9260**	**0.0036**	**0.0479**	**-1.8983**	**0.0416**	**0.4079**	**0.0599**	**0.0063**	**0.0134**	**0.0020**	**0.0781**	**1.47**	**0.41**	**4.41**	**3.27**
**FBgn0260858**	***Ykt6***	**Autophagy/Phagocytosis: Phagosome internalization; autophagosome expansion/fusion with lysosome**	**2.8690**	**-2.1510**	**0.0012**	**0.0271**	**-0.6169**	**0.1664**	**0.4079**	**0.5516**	**0.0003**	**0.0013**	**0.0270**	**0.0782**	**5.88**	**6.94**	**45.59**	**51.02**
FBgn0039705	*Atg16*	Autophagy/Phagocytosis: Phagosome maturation; autophagosome expansion	5.9160	-1.2470	0.0814	0.1579	-1.7148	0.0552	0.4079	0.1303	**0.0000**	**0.0000**	**0.0100**	0.0781	80.88	77.96	**7.35**	**4.90**
FBgn0010435	*emp*	Phagocytosis: Recognition—receptor	4.4820	-1.1770	0.1058	0.1811	-1.5438	0.0656	0.4079	0.2024	**0.0132**	**0.0241**	**0.0160**	0.0781	92.65	89.39	**5.88**	**3.67**
FBgn0014011	*Rac2*	Phagocytosis: Internalization	5.3780	-0.8280	0.2168	0.2634	-1.5064	0.0666	0.4079	0.0452	**0.0435**	0.0662	**0.0280**	0.0782	60.29	60.41	**8.82**	5.71
FBgn0031969	*pes*	Phagocytosis: Recognition—receptor	15.4180	-1.2590	0.0820	0.1584	-0.1963	0.2664	0.4152	0.0143	**0.0019**	**0.0051**	**0.0300**	0.0782	**4.41**	5.71	76.47	76.33
***D*. *simulans***																		
**Gene ID**	**Gene name**	**Function**	**θ**_**w**_	**TajD**	**TajD pval**	**TajD pval corrected**	**nFWH**	**nFWH pval**	**nFWH pval corrected**	**EW**	**EW pval**	**EW pval corrected**	**DHEW pval**	**DHEW pval corrected**	**TajD rank**	**TajD rank against null**	**nFWH rank**	**nFWH rank against null**
FBgn0191479	*Rab1*	Autophagy/Phagocytosis: Fusion with lysosome	1.5723	-1.4054	0.0766	0.2167	-0.8680	0.1365	0.7198	0.3673	0.0922	1.0000	**0.0078**	0.8734	12.31	11.64	**6.15**	**3.02**
FBgn0191695	*Atg8b*	Autophagy/Phagocytosis: Phagocytosis internalization; autophagosome expansion	2.8301	-1.6322	**0.0405**	0.2167	-0.8833	0.1246	0.7198	0.2857	**0.0183**	1.0000	**0.0117**	0.8734	10.77	11.64	**1.54**	**2.16**
FBgn0181823	*Vamp7*	Autophagy/Phagocytosis: Fusion with lysosome	1.8867	-1.9589	**0.0222**	0.2167	-0.7603	0.1454	0.7198	0.4388	**0.0242**	1.0000	**0.0197**	0.8734	**1.54**	**0.86**	**7.69**	**3.02**
FBgn0185198	*Rac1*	Phagocytosis: Internalization	2.8301	-1.9303	**0.0186**	0.2167	-0.3022	0.2352	0.7198	0.2347	0.0930	1.0000	**0.0450**	0.8734	**6.15**	6.47	26.15	22.41

θ_w_, Watterson’s *θ*; TajD, Tajima’s *D*; nFWH, normalized Fay and Wu’s *H*; EW, Ewens-Watterson statistic; DHEW, *DHEW* compound statistic P-value; TajD/nFWH/EW/DHEW pval corrected, P-values with multiple testing correction. For each focal-control pair, comparison score was calculated for Tajima’s *D* and Fay and Wu’s *H* and the scores were ranked from largest to smallest in the ‘rank’ column. Random assignment of a control gene to be “focal” and re-calculating the ranks of comparison scores created a distribution of ranks. The ranks of comparison scores from true coupling of focal and control genes compared to this distribution are listed in the ‘rank against null’ column.

**Table 3:** Genes that show significant and nominal evidence of amino acid divergence on the *D*. *melanogaster lineage* and *on the D*. *simulans* lineage.

***D*. *melanogaster***												
**Gene ID**	**Gene Name**	**Pn**	**Dn**	**Ps**	**Ds**	**MKcodons**	**Function**	**FETpval**	**FET corrected**	**DoS**	**DoS rank**	**DoS rank against null rank**
FBgn0260935	Ird1	30	1	47	59	1119	Autophagy/Phagocytosis: Phagosome maturation; autophagosome nucleation	**<0.001**	**<0.001**	-0.372943723	17 (26.15%)	39 (16.46%)
***D*. *simulans***												
**Gene ID**	**Gene Name**	**Pn**	**Dn**	**Ps**	**Ds**	**MKcodons**	**Function**	**FETpval**	**FET corrected**	**DoS**	**DoS rank**	**DoS rank against null rank**
**FBgn0189637**	***gb***	**4**	**7**	**53**	**8**	**447**	**Phagocytosis: Phagosome maturation**	**0.001**	**0.047**	**0.396**	**51 (100%)**	**179 (95.21%)**
FBgn0187055	*polyph*	3	4	47	5	320	Phagocytosis: Phagosome maturation	**0.008**	0.138	0.384	**47 (92.16%)**	173 (92.02%)
FBgn0182861	*scb*	11	12	75	21	814	Phagocytosis: Recognition—receptor	**0.008**	0.138	0.236	**49 (96.08%)**	174 (92.55%)
FBgn0045586	*Rbsn-5*	4	4	45	4	397	Phagocytosis: Phagosome maturation	**0.010**	0.142	0.418	**50 (98.04%)**	178 (94.68%)

Pn, the number of non-synonymous polymorphisms; Dn, the number of non-synonymous substitutions; Ps, the number of synonymous polymorphisms; Ds, the number of synonymous substitutions; MKcodons, the total number of codons subjected to the MK test; FETpval, P-value from Fisher’s exact test; FET corrected, P-value from Fisher’s exact test with multiple testing correction; DoS, direction of selection; For each focal-control pair, comparison score was calculated for DoS and the scores were ranked from largest to smallest in the ‘rank’ column. Random assignment of a control gene to be “focal” and re-calculating the ranks of comparison scores created a distribution of ranks. The ranks of comparison scores from true coupling of focal and control genes compared to this distribution are listed in the ‘rank against null’ column.

### Data

The sequences and the scripts to process them are available in https://github.com/imjoohyu.

## Results

### Survey of autophagy and phagocytosis genes in *D*. *melanogaster*

#### Autophagy and phagocytosis genes as a whole

To examine whether autophagy and phagocytosis genes exhibit any signature of recent positive selection, we calculated the following summary statistics for each focal gene and its corresponding control genes: Watterson’s estimator (*θ*_*w*_), Tajima’s *D* (TajD), normalized Fay and Wu’s *H* (nFWH), Ewens-Watterson’s homozygosity (*EW*), and the compound test statistic *DHEW*. For analyses of longer-term molecular evolution, we inferred the ancestral state of each substitution using *D*. *yakuba* and *D*. *simulans* as outgroups and assuming strict parsimony with no reverse or convergent mutation. We then compared polymorphism and divergence at synonymous and non-synonymous sites using the MK test [41] and calculated the Direction of Selection (DoS) on each gene. To determine whether the combined group of all autophagy and phagocytosis genes shows evidence of recent selection or recurrent adaptive evolution, we explored whether the population genetic statistics of these genes are statistically significantly different from their respective control genes by calculating the mean of comparison scores for each focal-control gene pairing for each statistic (Table 1). The only observed significant difference was in the *EW* statistic between autophagy and phagocytosis genes and their control genes in *D*. *melanogaster* (t-test p = 0.022, Wilcox test p = 0.055), pointing to the enrichment of low-frequency derived alleles with an increase in haplotype homozygosity in the focal genes. Randomly assigning control genes to serve as “focal” genes and repeating the analysis removed this significant difference, indicating that the pattern of reduced polymorphism is unique to the autophagy and phagocytosis genes. Removing 10% of the genes with the lowest individual P-values and repeating the analysis still preserved the pattern. Thus, this statistically significant difference between *EW* values of focal and control genes is a cumulative effect over the full set of autophagy and phagocytosis genes and is not driven by a few genes that are strongly divergent from the null expectation. Contrary to what was observed in autophagy and phagocytosis genes, a set of humoral signaling genes and a set of recognition genes did not differ significantly from their control genes in any of the statistics.

#### Individual autophagy and phagocytosis genes

We tested for signatures of recent selection in individual genes and only *Atg14* and *Ykt6* in *D*. *melanogaster* had significant test statistics after false discovery correction and extreme comparison scores (Table 2). *Atg14* (*D* = -1.926, p = 0.048, *EW* = 0.06, p = 0.013) encodes an endoplasmic reticulum (ER) protein that helps autophagosomes nucleate [44]. The *Atg14* comparison score for *D* was the largest and for *H* was in the top 5% among 71 gene pairs surveyed (Table 2). These observations can be attributed to natural selection because effects of chromosomal position or demographic history would also be expected to impact the control genes. *Ykt6*, a SNARE protein involved in internalization of particles during phagocytosis and expansion of the autophagosome membrane [45, 46], showed significant P-values for *D* and *EW* (*D* = -2.151, p = 0.027, *EW* = 0.552, p = 0.001). The *D* comparison score for *Ykt6* was the 5th largest in the *D*. *melanogaster* data set, illustrating the enrichment of low-frequency variants specifically at this locus (Table 2). In addition, CD36 scavenger receptors *emp* and *pes* [47–49], autophagy gene *Atg16*, and phagocytosis gene, *Rac2*, showed significant *EW* and *DHEW* statistics but did not meet the multiple testing correction threshold (Table 1). Next, we looked for evidence of recurrent adaptive evolution in individual autophagy and phagocytosis genes. While *Ird1* showed significant MK test results even after multiple testing correction (<0.05), the DoS value for each of these genes was negative and was not drastically different from its control genes according to the DoS comparison score. This result indicates that slightly deleterious mutations are segregating at these loci (S1 Table) and provides no support for recurrent adaptive amino acid substitution.

### Survey of autophagy and phagocytosis genes in *D*. *simulans*

#### Autophagy and phagocytosis genes as a whole

We tested whether phagocytosis and autophagy pathways as a whole show distinct patterns of sequence evolution compared to control genes in *D*. *simulans*. As was the case in *D*. *melanogaster*, the significant difference between *D*. *simulans* phagocytosis and autophagy genes and their control genes was seen in the *EW* statistic (t-test p = 0.003, Wilcox test p = 0.005, Table 1). Randomly re-assigning a control gene to be “focal” eliminated this significant difference, suggesting that the observed pattern is attributable to phagocytosis and autophagy genes. Again, consistent with the patterns in *D*. *melanogaster*, the statistically significant difference between *EW* values of focal and control genes is not due to a few divergent genes, but rather is a pattern seen across all genes. While humoral immune signaling genes in *D*. *simulans* did not differ significantly from their control genes in any of the statistics, the DoS statistic for recognition genes showed deviation from that of respective control genes according to the t-test (p = 0.005; Table 1). This trend was largely driven by the fact that only three gene pairs existed in this category and that the DoS values of four out of ten corresponding control genes are highly positive (>0.2).

#### Individual autophagy and phagocytosis genes

We tested for signatures of recent selection in individual genes in *D*. *simulans*. While four genes, *Rab1*, *Atg8b*, *Vamp7* and *Rac1* (the first three are involved in both phagocytosis and autophagy pathways, whereas the last is implicated only in phagocytosis) showed nominally significant *D*, *EW*, and *DHEW* statistics, none remained significant after the multiple testing correction. When we compared polymorphism and divergence at synonymous and non-synonymous sites using the MK test to test for recurrent adaptive amino acid substitution along the *D*. *simulans* lineage, we found that only *gb* had significant MK test results after the multiple testing correction and a positive DoS value (MK corrected FET p = 0.046, DoS = 0.396, Table 3) The DoS comparison score for *gb* also ranked the highest when compared to other gene pairs (Table 3). *gb* encodes a glutamate transporter that regulates the extracellular glutamate levels in the nervous system [50] and controls internal ROS and to promote phagosome maturation [51]. The other glutamate transporter *polyph* [50, 51], as well as phagocytic receptors *pes* and *scb* [49, 52], and *Rbsn-5*, which facilitates phagosome maturation [2], also showed nominally significant MK results that did not meet the multiple testing correction threshold (Table 3). We identified no autophagy genes evolving with an elevated rate of amino acid substitution along the *D*. *simulans* lineage.

## Discussion

Dynamic conflict between hosts and pathogens may result in co-evolutionary adaptation in host genes, resulting in signatures of positive selection. Previous work to understand the molecular evolutionary patterns of immune genes in *Drosophila* has enriched our understanding of how the innate immune system has evolved. However, most population genetic studies on innate immunity have so far focused on the humoral immune response genes and phagocytic receptor genes, so the evolution of most of cellular immunity remains to be understood. In this study, we examined molecular evolutionary patterns of autophagy and phagocytosis genes in *D*. *melanogaster* and *D*. *simulans*. We found that phagocytosis and autophagy pathways as a whole showed an elevated level of haplotype homozygosity in both species, suggesting that genes in these pathways demonstrate small indications of adaptation that collectively result in a statistically measurable deviation from neutrality. The *EW* test statistic is more powerful for detecting a very recent selective sweep compares to other tests [53]. The aggregate significance of the *EW* test would therefore seem to indicate that many autophagy and phagocytosis genes have been targets of recent sweeps, but that the pattern has not persisted over enough evolutionary time to leave signatures detectable by other test statistics. It is unclear what biological scenario would trigger a widespread set of recent sweeps, although ecological shift or invasion of a novel pathogen is possible. We identified several individual genes that exhibit indications of positive selection, although only a subset of them were statistically significant after controlling for multiple testing.

### Positive selection on autophagy genes

We looked for signatures of natural selection in autophagy genes, which have both defensive and housekeeping roles. Besides removal of pathogens, autophagy degrades damaged host proteins and organelles to recycle nutrients during stressful conditions, such as starvation [5]. The core autophagy machinery is conserved from yeast to higher eukaryotes [4]. The role of autophagy in maintaining organismal homeostasis could act as a constraint on adaptation in response to pathogen pressure, which might explain why we did not see pervasive signatures of positive selection in autophagy genes of either *D*. *melanogaster* or *D*. *simulans*. However, we did identify signatures of recent positive selection in *Atg14* and *Ykt6*, which remained significant after multiple testing correction in *D*. *melanogaster*. *Atg14* is conserved in both *Drosophila* and mammals and is involved in the nucleation of phagosphore membrane [3]. Upon infecting mouse and human cell lines, the intracellular bacterium *Brucella abortus* forms a *Brucella*-containing vacuole using the host proteins Atg14 and Beclin-1, the mammalian homolog of Atg6, in order to be trafficked to the ER where the bacterium proliferates [54]. Similar interactions with pathogens may have led to *Atg14* adapting to play a more specific role in immune defense and to our observed evolutionary pattern in *D*. *melanogaster*. While the test statistics of *Atg16* did not meet the multiple testing correction threshold, its nominally significant test statistics indicate that it may have experienced a strong selective pressure because it is responsible for removing both intra- and extracellular pathogens [54].

### Positive selection on glutamate transporters

We also identified a glutamate transporter gene, *gb*, to have elevated amino acid sequence divergence on the *D*. *simulans* lineage (Table 3). *gb* encodes a transporter that controls the extracellular glutamate levels in the nervous system [51]. When *gb* is mutated, glutamate level in the hemolymph is reduced, synthesis of glutathione (a major antioxidant) is disrupted, and the intracellular ROS is increased [55], leading to a failure in producing mature phagosomes and in phagocytosis of *Staphylococcus aureus* in *Drosophila* [51]. Another putative glutamate transporter gene *polyph* that also plays a role in regulating glutamate level showed a significant MK result prior to multiple testing correction [51]. Although it is unknown whether these proteins physically interact with each other, both genes are expressed in *Drosophila* blood cells and share a function [51]. Due to this shared function and evolutionary pattern, it is tempting to speculate that positive selection may be acting on these proteins together. A host protein that evolves to escape pathogen interference may also evolve away from its native function within the host. Thus, compensatory mutation in interacting proteins that restore full function could become adaptive [56].

### Positive selection on recognition genes in phagocytosis

Opsonins and phagocytic receptors have been hypothesized to evolve under host-pathogen co-evolution because they directly bind to molecules from pathogens in order to promote phagocytosis [57–61]. Previous research reported that *emp*, *pes*, and *scb*, which encode phagocytic receptors that bind to pathogens bore evidence of recent and adaptive evolution in *Drosophila* [58, 59, 62]. *TepII*, *TepIV* (opsonin), *Sr-CI* (scavenger receptor) and nimrod genes (*NimC1*, *NimB4*) also show an excess of non-synonymous fixations between *D*. *melanogaster* and *D*. *simulans* in [57–59]. While *emp* and *pes* in *D*. *melanogaster* and *pes* and *scb* in *D*. *simulans* in this study showed some indication of selection, the test statistics were not significant after multiple testing correction. These distinctions between previous studies and the present one may be due to differences in methods since our method employed a composite method in addition to typical test statistics used in the field to determine significance.

### No parallel adaptation between *D*. *melanogaster* and *D*. *simulans*

One objective of this study was to evaluate whether the same genes in *D*. *melanogaster* and *D*. *simulans* show similar patterns of non-neutral evolution as a test for parallel adaptation. However, we did not find evidence for parallel adaptation at the gene level. While *D*. *melanogaster* and *D*. *simulans* are closely related species, the locations in which they were collected, their ecology, and their demographic histories differ [63]. While *D*. *melanogaster* and *D*. *simulans* are known to share some viruses, such as the Galbut Virus [64], there is no information about whether they share any bacterial pathogens in the wild and it is reasonable to suppose they may generally be exposed to different pathogens because of difference in micro- or macro-ecology. If these distinct pathogens interact differently with the two host species, then parallel adaptation may be unlikely. It is important to note that detection of parallel evolution relies on the detection of statistically significant patterns in both species. Thus, they may be under-detected if the analysis has low statistical power. In our particular study, the number of lines sampled from each species differed, so the power to detect selection was not equivalent across the species. Nevertheless, our finding is consistent with previous reports suggesting that parallel adaption is not common. For example, a previous study noted that while selective sweeps affect antiviral pathways in many insect species, the affected genes varied considerably across species [65]. Another study reported that a humoral signaling gene, *Relish*, known to have undergone adaptive divergence in *D*. *simulans*, did not exhibit as strong of evidence of natural selection in three other sister species [66].

### Soft sweeps and balancing selection

Our study mainly focused on detecting strong signatures of natural selection, as would be expected from hard selective sweeps favoring novel mutations [67], and on recurrent adaptive amino acid substitutions in a gene [41, 68]. These are the signatures expected from classic arms race model, which was our core biological hypothesis. We have less power to detect other forms of adaptation that might act on immune system genes, including soft sweeps of adaptation from standing genetic variation and balancing selection. Soft sweeps refer to a mode of adaptation where multiple distinguishable adaptive alleles are present in the population at the same time [69]. Soft sweeps may be common in *D*. *melanogaster* [70, 71] but are not easily detected by frequency-based statistics such as Tajima’s *D* or Fay and Wu’s *H* because genetic diversity is not as severely reduced when the selected site is on multiple haplotype backgrounds or is not driven to complete fixation [72]. A previous study that looked for genes evolving via soft sweeps in *D*. *melanogaster* [71] showed evidence for soft sweeps in chromosomal regions that include *Vamp7* and *Sr-CII*, which did not show statistically significant results in our study, although the precise targets of these sweeps remain unidentified.

In the context of host-pathogen interactions, balancing selection can theoretically be generated if polymorphisms arise at co-evolving loci of both hosts and pathogens and two or more alleles are maintained at static intermediate or oscillating frequencies, or if alleles are costly in the absence of infection so cannot be driven to fixation [11, 73–75]. The genomic signatures of balancing selection can be detected in organisms whose breeding structures or population sizes result in linkage disequilibrium that extends over long physical stretches of chromosomes, such as *Arabidopsis* [73, 76] and humans [77, 78]. However, it is much more difficult to detect balancing selection in organisms like *D*. *melanogaster* that have large population sizes and high rates of recombination [79, 80]. Therefore, we cannot rule out the possibility that some components of autophagy and phagocytosis systems in *Drosophila* may have evolved under undetected balancing selection.

## Supporting information

S1 Fig Correlation between recombination rates of focal genes and recombination rates of corresponding control genes (L). Recombination rates of focal genes (x-axis) were plotted against mean recombination rates of respective control genes (y-axis) based on the study of [27]. (R) Recombination rates of focal genes (x-axis) were plotted against mean recombination rates of respective control genes (y-axis) based on the study of [28].
(TIF)

S1 Table List of all genes in *D*. *melanogaster* surveyed for population genetic analysis and the results of the analysis. (XLSX)

S2 Table List of all genes in *D*. *simulans* surveyed for population genetic analysis and the results of the analysis. (XLSX)
