# Supplementary figures and images for: Population genetic analysis of autophagy and phagocytosis genes in Drosophila melanogaster and D. simulans
Source: PLoS One. 2018 Oct 3;13(10):e0205024. doi: 10.1371/journal.pone.0205024 (PMC6169979; doi:10.1371/journal.pone.0205024)

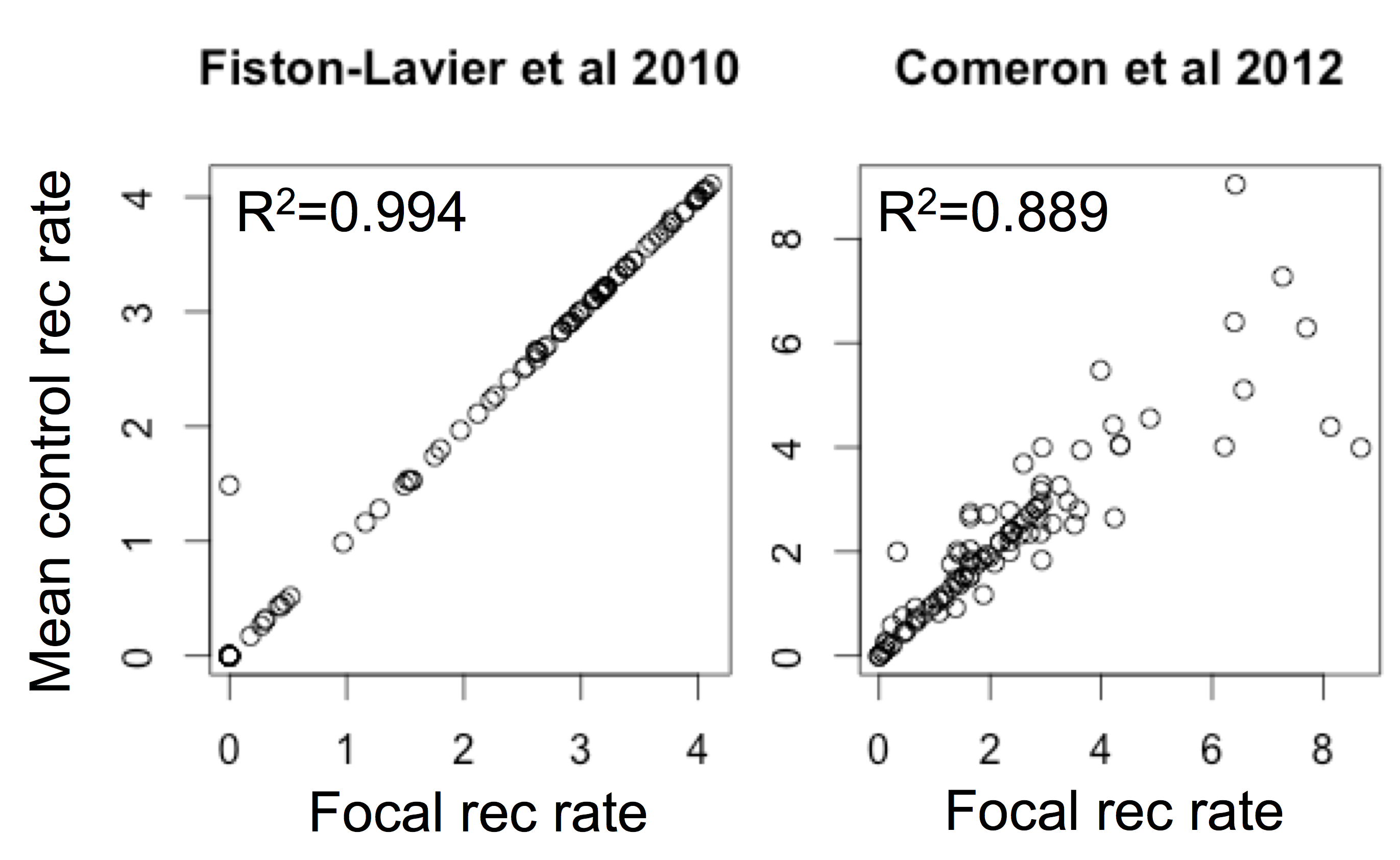

Supplement: S1 Fig — Recombination rates of focal genes (x-axis) were plotted against mean recombination rates of respective control genes (y-axis) based on the study of [27]. (R) Recombination rates of focal genes (x-axis) were plotted against mean recombination rates of respective control genes (y-axis) based on the study of [28]. (TIF) [file pone.0205024.s001.tif]
